# The X-linked tumor suppressor TSPX downregulates cancer-drivers/oncogenes in prostate cancer in a C-terminal acidic domain dependent manner

**DOI:** 10.18632/oncotarget.26673

**Published:** 2019-02-19

**Authors:** Tatsuo Kido, Yunmin Li, Yuichiro Tanaka, Rajvir Dahiya, Yun-Fai Chris Lau

**Affiliations:** ^1^ Division of Cell and Developmental Genetics, Department of Medicine, Veterans Affairs Medical Center, San Francisco, California, USA; ^2^ Institute for Human Genetics, University of California, San Francisco, California, USA; ^3^ Department of Urology, Veterans Affairs Medical Center, San Francisco and University of California San Francisco, San Francisco, California, USA

**Keywords:** prostate cancer, tumor suppressor, oncogene, transcriptome analyses, TCGA

## Abstract

TSPX is a tumor suppressor gene located at Xp11.22, a prostate cancer susceptibility locus. It is ubiquitously expressed in most tissues but frequently downregulated in various cancers, including lung, brain, liver and prostate cancers. The C-terminal acidic domain (CAD) of TSPX is crucial for the tumor suppressor functions, such as inhibition of cyclin B/CDK1 phosphorylation and androgen receptor transactivation. Currently, the exact role of the TSPX CAD in transcriptional regulation of downstream genes is still uncertain. Using different variants of TSPX, we showed that overexpression of either TSPX, that harbors a CAD, or a CAD-truncated variant (TSPX[∆C]) drastically retarded cell proliferation in a prostate cancer cell line LNCaP, but cell death was induced only by overexpression of TSPX. Transcriptome analyses showed that TSPX or TSPX[∆C] overexpression downregulated multiple cancer-drivers/oncogenes, including MYC and MYB, in a CAD-dependent manner and upregulated various tumor suppressors in a CAD-independent manner. Datamining of transcriptomes of prostate cancer specimens in the Cancer Genome Atlas (TCGA) dataset confirmed the negative correlation between the expression level of TSPX and those of MYC and MYB in clinical prostate cancer, thereby supporting the hypothesis that the CAD of TSPX plays an important role in suppression of cancer-drivers/oncogenes in prostatic oncogenesis.

## INTRODUCTION

Prostate cancer is one of the leading cancer in the world [1]; more than 200,000 cases are newly diagnosed every year in the United States [2]. The molecular mechanisms associated with prostate cancer development are complex and still largely unknown, although various susceptibility loci/SNPs have been associated with either the familial or sporadic prostate cancer [3, 4]. Recent studies have suggested the involvements of various X-linked tumor suppressors in the prostate cancer development [5–7]. Since men have only a single X-chromosome, any inactivating mutation(s) on the X-linked tumor suppressor genes could predispose men for cancer development [5–7]. Indeed, a prostate cancer susceptibility locus (loci) has been mapped on Xp11.2 associated with the SNP rs5945572 [8], adjacent of which multiple tumor suppressor genes, including FOXP3 and AMER1 (also known as WTX and FAM123B), are located [7, 9, 10]. FOXP3 is frequently downregulated or inactivated by a mutation(s) in prostate cancer, and reactivation of FOXP3 expression suppresses cancer growth [10, 11]. Mutations in the AMER1 gene are frequently detected in male colorectal cancer [12], although its status with prostate cancer is uncertain. Hence, alterations of X-linked genes, particularly tumor suppressors, could play important roles in prostate cancer initiation and progression.

The TSPY homologue on the X chromosome (TSPX, also known as CDA1, DENTT, and TSPYL2) is another tumor suppressor gene located at the Xp11.2 locus. TSPX is ubiquitously expressed in normal tissues (research reports [13, 14], and data portals; GTEx [15] and BioGPS [16]), but it is frequently downregulated in various types of cancer, including lung cancer, glioma, and liver cancer [17–19]. It has been demonstrated that overexpression of TSPX in a lung carcinoma cell line A549 and a cervical cancer cell line HeLa resulted in retardation of cell proliferation [17, 20]. TSPX upregulated CDKN1A gene by activating TP53-pathway in HeLa cells [20], and/or by suppressing NTRK3 proto-oncogene (also known as Trk-C) in A549 cells via the TGFβ-SMAD pathway [21]. At present, its role(s) in prostate cancer has not been fully investigated, although our previous study demonstrated that TSPX could inhibit the transactivation of androgen receptor (AR) on its target genes [22]. Since the functions of tumor suppressors could be cell type specific [23–25], it is important to examine the role(s) of TSPX in prostate cancer development and the associated mechanism(s) in oncogenesis.

TSPX is a homologue of the Y-encoded proto-oncoprotein TSPY that exacerbates cancer cell growth *in vitro* and *in vivo* [26]. TSPX and TSPY harbor a conserved domain, termed SET/NAP domain, initially identified in the oncoprotein, SE translocation (SET, also known as TAF-Iβ) and the nucleosome assembly proteins (NAPs), but diverged at the flanking regions [27, 28]. The TSPX protein harbors 3 major domains, (i) a proline-rich domain in the N-terminus, (ii) the centrally located SET/NAP-domain and (iii) a long Asp/Glu-rich acidic domain in the C-terminus (hereby designated as C-terminal acidic domain, CAD) [27, 28]. Although TSPX and TSPY genes evolved from a common ancestral gene, only TSPX possesses a proline-rich domain and the CAD [29, 30]. Significantly, we have demonstrated that the CAD is primarily responsible for contrasting functions between TSPX and TSPY. For example, both proteins interact with cyclin B via their respective SET/NAP-domain, but TSPY stimulates while TSPX inhibits the kinase activity of cyclin B/CDK1 complex [28]. The inhibitory domain has been mapped to the CAD of TSPX [28]. Further, we recently demonstrated that TSPX could interact and inhibit the transactivation activity of androgen receptor (AR) in a CAD dependent manner. TSPX overexpression represses the expression of AR target genes, including KLK2 and KLK3, in a prostate cancer cell line LNCaP [22]. Since AR plays fundamental roles in the initiation and progression of prostate cancer [31, 32], TSPX might work as a modular for androgen and AR activities in the prostate. TSPX is primarily located in the nucleus, and presumed to play a role in transcription. Hence, understanding the roles of TSPX, particularly its CAD, in general transcriptional regulation of gene expression will be essential to determine its contributions to prostatic oncogenesis and cancer progression.

To explore the above issues, we have examined the effects of overexpression of the full length and variant versions of TSPX in the prostate cancer cell line LNCaP, and determined the respective effects in cell viability, morphology and gene expression patterns using RNA-Seq strategy. The expression patterns were then compared with those of clinical prostate cancer specimens with high or low TSPX expression from the Cancer Genome Atlas (TCGA) dataset [33]. Our results showed that overexpression of TSPX and/or its variants affected cell proliferation, morphology and viability. Transcriptome analyses demonstrated that the expression levels of various cancer-drivers/oncogenes, including MYC and MYB, were negatively correlated with that of TSPX in both LNCaP cells and clinical prostate cancer samples. Specifically, the expressions of MYC and MYB were suppressed by TSPX in LNCaP cells in a CAD-dependent manner. Our findings suggest that TSPX is a crucial X-linked tumor suppressor in prostate cancer and its CAD plays important roles in the downregulation of multiple cancer-drivers/oncogenes, and are novel targets for diagnosis and clinical treatment of prostate cancer.

## RESULTS

### TSPX is frequently downregulated in prostate cancer

**Figure 1:**
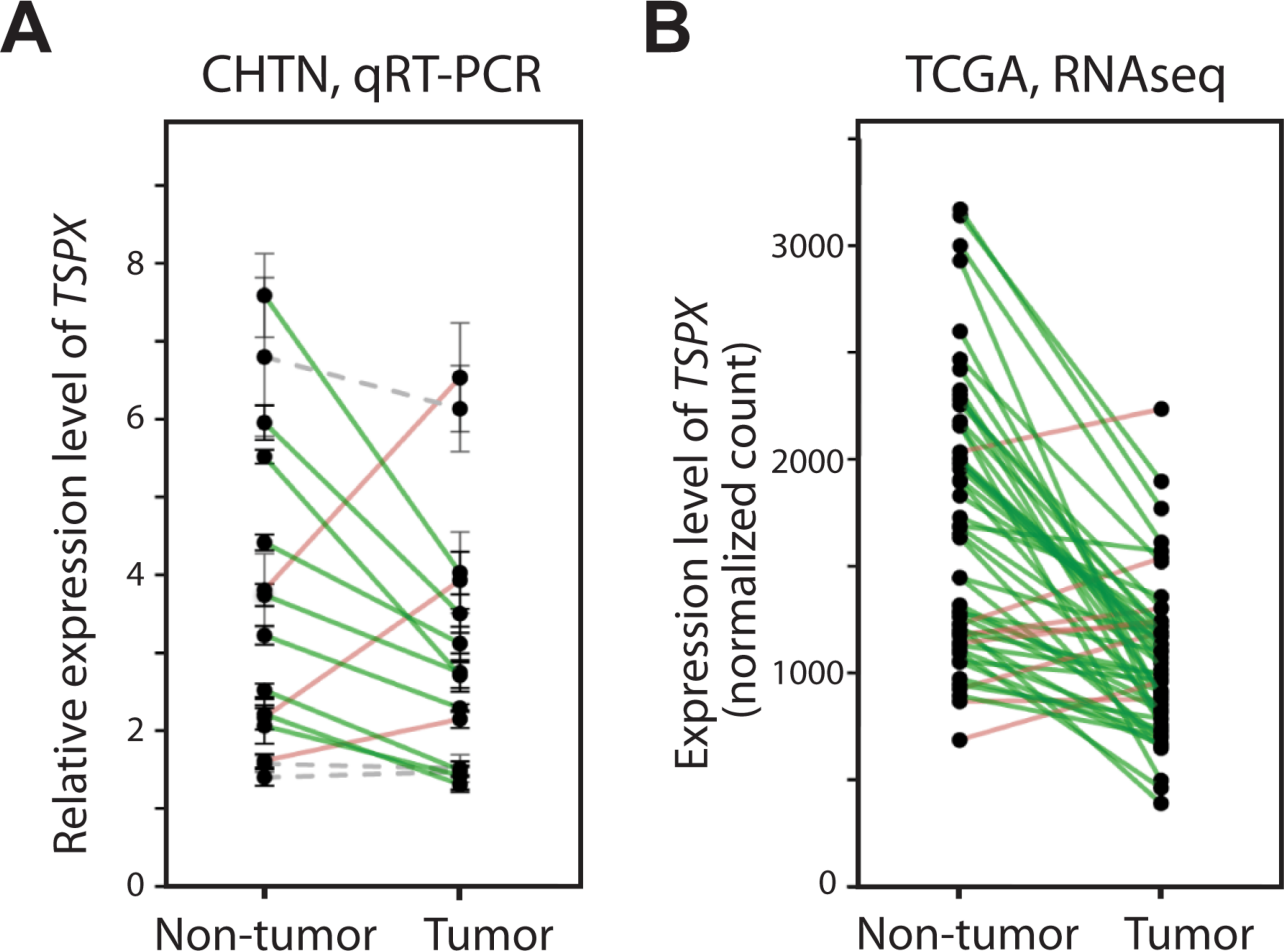
The expression level of TSPX was frequently down-regulated in clinical prostate cancer (**A**) Results of real-time qRT-PCR analysis of the TSPX expression levels in 15 prostate cancer tumor/non-tumor paired samples obtained from the Cooperative Human Tissue Network (CHTN). Expression values were normalized against GAPDH, and samples from the same patient are linked with a solid line respectively. Green indicate down-regulated cases, and red indicate up-regulated cases. Gray dotted lines indicate no significant change between non-tumor and tumor samples (*P* > 0.05). (**B**) Results of datamining of the RNA-Seq dataset from 52 prostate cancer tumor/non-tumor paired samples from the Cancer Genome Atlas (TCGA). Expression values (normalized count values) were plotted, and samples from the same patient are linked with a straight line as described above.

To explore the expression patterns of TSPX in prostate cancer, we had analyzed its expression levels in 15 paired samples of prostate cancer (T) and their adjacent non-tumor tissue (NT) by quantitative RT-PCR (qRT-PCR). The result showed that TSPX was significantly downregulated in 9 cases (60%), while it was upregulated in 3 cases (20%) (Figure 1A and Table 1). Although the sample size was small to obtain a statistical significance, a general observation is that TSPX tends to be downregulated in prostate cancer. To verify the preliminary results of qRT-PCR analysis, we had datamined the RNA-Seq gene expression data of clinical prostate cancer samples downloaded from the Cancer Genome Atlas (TCGA) [33]. Of the 52 cases with tumor and non-tumor paired samples, TSPX was downregulated in 44 cases (83%) of prostate cancer, as compared to the adjacent normal specimens, and was up-regulated in 6 cases (11%) (Figure 1B and Table 1), indicating that TSPX was significantly downregulated in prostate cancer of TCGA dataset (Wilcoxon matched pair test *P*-value < 0.0001). These observations confirm those of qRT-PCR analysis and suggest that TSPX expression might be involved in prostate cancer development.

**Table 1:** Summary of the results of qRT-PCR analysis of CHTN RNA samples and data-mining of transcriptomes of TCGA datasets on paired tumor and non-tumor specimens (see Figure 1)

Sample	NT > T	NT = T	NT < T	Total (cases)	*P*-value
CHTN	9 (60%)	3 (20%)	3 (20%)	15	0.1248
TCGA	44 (85%)		8 (15%)	52	<0.0001

NT > T: the TSPX expression level was lower in tumor sample (T) than the corresponding non-tumor sample (NT); NT = T: the TSPX expression level was similar between T and NT; NT < T: the TSPX expression level was higher in T than NT; *P*-value: Wilcoxon matched pair test *P*-value.

### Overexpression of TSPX induces morphological change and cell death in LNCaP cells

**Figure 2:**
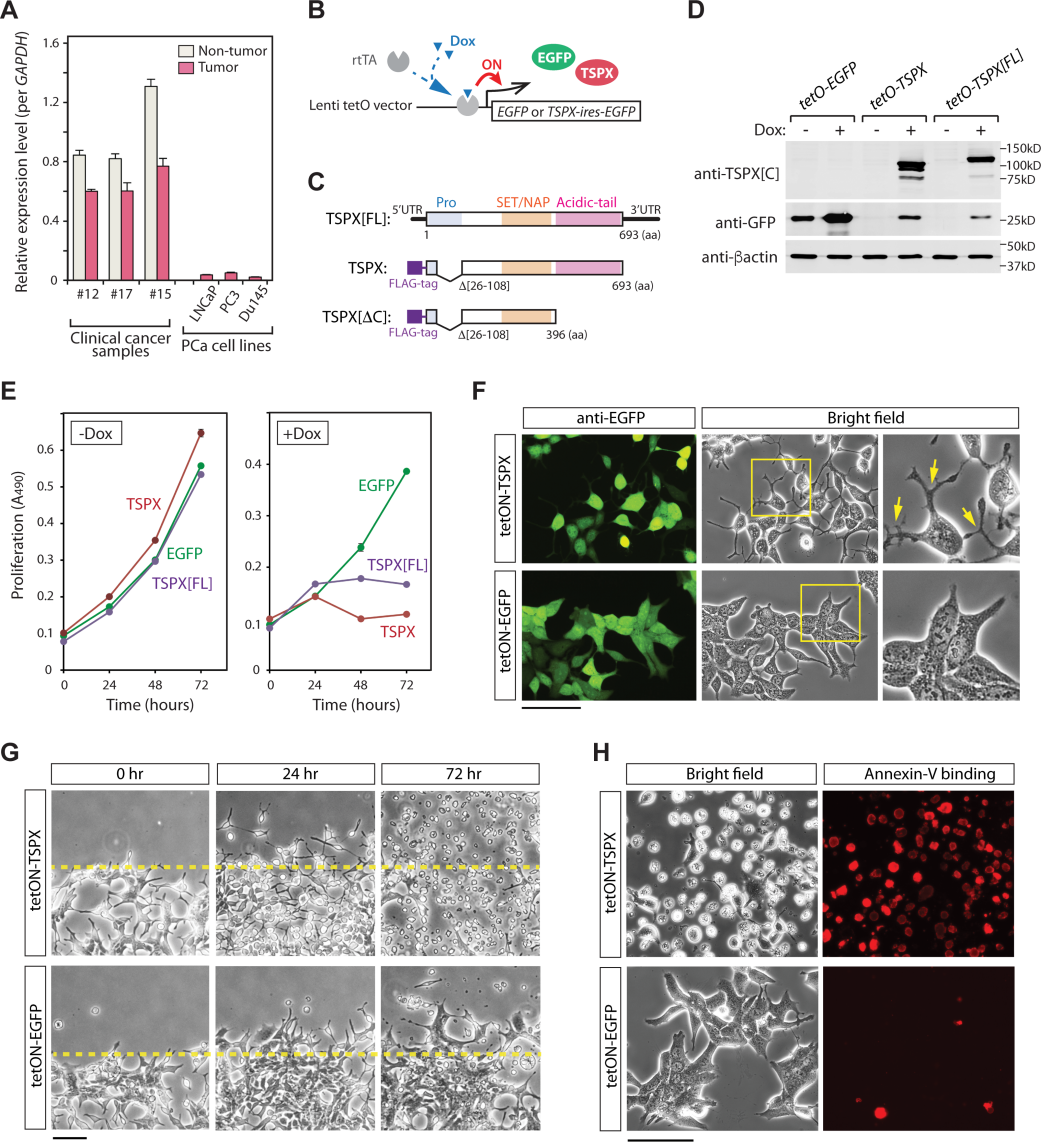
Overexpression of TSPX caused morphological changes and cell-death in LNCaP cells (**A**) Comparison of the expression levels of TSPX among clinical prostate cancer samples and prostate cancer cell lines, LNCaP, PC3 and DU145. The expression levels were measured by qRT-PCR and normalized against GAPDH. (**B**) A schematic diagram of tet-ON system. In the presence of doxycycline (Dox), a transactivator rtTA is recruited on to the promoter region, and turns on the expression of transgene. (**C**) A diagrammatic illustration of transgene constructs for TSPX[FL], TSPX, and TSPX[ΔC]. (**D**) Western blot confirmed the expressions of EGFP and TSPX variants in the respective transduced LNCaP cells. β-actin was used as an internal and loading control. (**E**) Cell proliferation assay showed that expression of TSPX[FL] or TSPX (+Dox) inhibited cell proliferation, as compared to non-expressors (-Dox) or EGFP alone. (**F**) Cell morphologies of LNCaP-tetON-TSPX and LNCaP-tetON-EGFP cells at 24 hours after transgene induction. The co-expression of EGFP was confirmed by immunofluorescence (green). The TSPX overexpression induced morphological changes to round shapes with dendrite-like protrusions in LNCaP cells (arrows), but not EGFP alone. Far right panels show magnified images of the boxed area in the middle panels. (**G**) Scratch tests for LNCaP-tetON-TSPX cells (top) and LNCaP-tetON-EGFP cells (bottom) under transgene induction conditions. The morphology of LNCaP-tetON-TSPX cells dramatically changed by 24 hours, and numerous cells detached from the growing surface by 72 hours. (**H**) Annexin-V binding assay at 48 hours showed that the detached LNCaP-tetON-TSPX cells were positively stained by Annexin-V conjugated with Alexa Fluor 594 (red), corresponding to dead or apoptotic cells. Scale bar= 100 μm in F and H, 200 μm in G.

To explore the consequences of high TSPX expression in prostate cancer, various metastatic prostate cancer cell lines, e.g. LNCaP, PC3, and DU145, were analyzed with qRT-PCR strategy. Our results showed that TSPX expression was remarkably lower in these established prostate cancer cell lines than those of the primary prostate tissues (Figure 2A). Since the prostatic cancer cell lines are highly proliferative, these observations supported the postulation that TSPX expression is inversely correlated with cell proliferation and hence prostatic oncogenesis. To examine the effects of TSPX in prostatic cancer cells, the LNCaP cells were transduced with the tet-ON lentiviral vector system expressing EGFP and TSPX variants, i.e. TSPX[FL] with a longest N-terminal region or TSPX with a shorter N-terminal region (Δ26-108aa), under the control of doxycycline (Dox) (Figure 2B, 2C). The resultant cells were designated as LNCaP-tetON-TSPX[FL] and LNCaP-tetON-TSPX, respectively. LNCaP-tetON-EGFP cells were used as a reference in these experiments. Western-blot analyses confirmed that the expressions of TSPX[FL] and FLAG-tagged TSPX were appropriately controlled by Dox treatment (Figure 2D). Cell proliferation assays were performed at 0, 24, 48 and 72 hours. Our results showed that both TSPX[FL] and TSPX completely suppressed cell proliferation by 48 hours after Dox administration (Figure 2E). At 24 hour of transgene induction, the both LNCaP-tetON-TSPX[FL] and LNCaP-tetON-TSPX cells took on various round-shaped morphologies with cellular protrusions (Figure 2F), suggesting that TSPX-overexpression could retard cell proliferation and induce cellular structural changes in LNCaP cells. Since TSPX lacks residue #26-108 at the N-terminus encompassing the proline-rich domain, the results also suggest that this domain is not critical for the cell proliferation and morphological functions.

Scratch tests showed that the overexpression of TSPX led to dramatic morphological changes (Figure 2G, 24 hr); and most cells detached from dish surface and floated in culture medium by 72 hours after Dox-induction (Figure 2G, 72 hr), as compared to tetON-EGFP control cells. An Annexin-V binding assay showed that the floating cells were apoptotic/dead cells positively stained by Annexin-V (Figure 2H, red-stained cells). These observations suggest that high-level TSPX expression greatly inhibits cell proliferation and viability.

### The C-terminal acidic domain of TSPX is crucial for induction of cell death and morphological change in LNCaP cells

**Figure 3:**
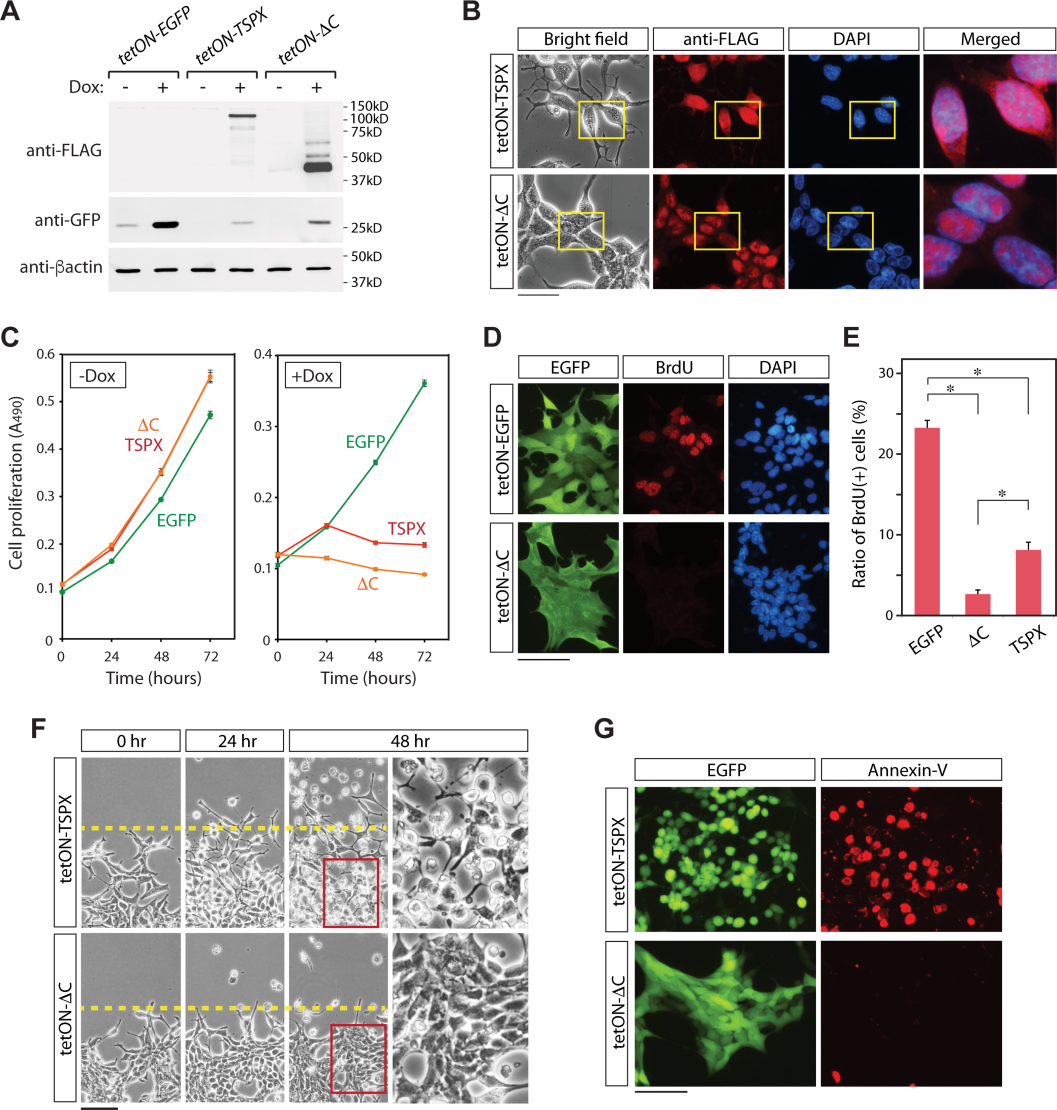
Effects of truncation of the CAD from TSPX in LNCaP cells (**A**) Western blots confirming the expressions of EGFP and FLAG-tagged TSPX variants in the respective transduced LNCaP cells. (**B**) Bright-field and immunofluorescence images of the FLAG-tagged TSPX variants (red), and DAPI staining (blue) in the respective transduced LNCaP cells at 24 hours after Dox-induction. Far right panels show the merged images of anti-FLAG staining and DAPI staining in the boxed areas. (**C**) Cell proliferation assay showed that overexpression of TSPX or TSPX[ΔC] (+Dox) inhibited cell proliferation, as compared to non-expressers (-Dox) or EGFP alone. (**D**) Immunofluorescence of the BrdU incorporation (red) for LNCaP-tetON-EGFP cells and LNCaP-tetON-ΔC cells showed that cells overexpressing TSPX[ΔC] lacked DNA synthetic activities. (**E**) Quantified results of the BrdU incorporation assay. Bars indicate the percentage of BrdU-positive cells in the LNCaP-tetON-EGFP, LNCaP-tetON-TSPX, and LNCaP-tetON-TSPX[ΔC] population respectively. An asterisk indicates significance at *P* < 0.01. (**F**) Time dependent changes of LNCaP-tetON-TSPX cells (top) and LNCaP-tetON-ΔC cells (bottom) after Dox-induction of respective transgenes. Bright-field images of the same area at the indicated time points are presented. Far right panels show magnified images of the boxed area in the middle panels. (**G**) Annexin-V binding assay (red) at 48 hours after Dox-induction showed that only LNCaP-tetON-TSPX cells were positive for this apoptotic marker, but not LNCaP-tetON-ΔC cells. EGFP green fluorescence in living cells are also presented on the left. Scale bar= 50 μm in B, 200 μm in E, 100 μm in F and G.

Our previous studies demonstrated that the C-terminal acidic domain (CAD) of TSPX is critical for its tumor suppressor functions, such as suppression of cyclin B/CDK1 phosphorylation activities [28], degradation of a HBV viral oncoprotein HBx [34], and inhibition of the androgen receptor (AR) transactivation [22]. To explore the potential role of the CAD in regulation of cell proliferation and cell death in LNCaP cells, we had transduced the LNCaP cells with a TSPX variant with a truncated CAD (TSPX[ΔC]), designated as LNCaP-tetON-ΔC cells, under the control of Dox (Figure 2B and 2C). Expression of TSPX[ΔC] in LNCaP-tetON-ΔC cells was confirmed by western-blot (Figure 3A). Immunocytochemical analyses showed that both TSPX and TSPX[ΔC] were predominantly localized in the nuclei (Figure 3B). Cell proliferation assays unexpectedly showed that the overexpression of TSPX[ΔC] could also suppressed cell proliferation in LNCaP cells, similarly to those expressing TSPX transgene (Figure 3C, ΔC). The BrdU incorporation assays performed at 24 hours after Dox-induction demonstrated that the ratio of BrdU positive cells was significantly lower in both LNCaP-tetON-ΔC cells and LNCaP-tetON-TSPX cells than LNCaP-tetON-EGFP reference cells with Dox-induction, suggesting that DNA synthetic activities were significantly repressed in TSPX or TSPX[ΔC] expressing cells (Figure 3D and 3E). However, overexpression of TSPX[ΔC] did not induce either morphological change or detachment from the growing surface (Figure 3F, tetON-ΔC). Further, LNCaP-tetON-ΔC cells were rarely stained by Annexin-V at 48 hours after Dox-induction (Figure 3G). These results suggest that TSPX could suppress cell proliferation, but its CAD is essential for induction of cell death and cellular morphological/structural changes in LNCaP cells.

### High-level TSPX affected the gene expression patterns related with cell-viability and cell morphology in LNCaP cells

**Figure 4:**
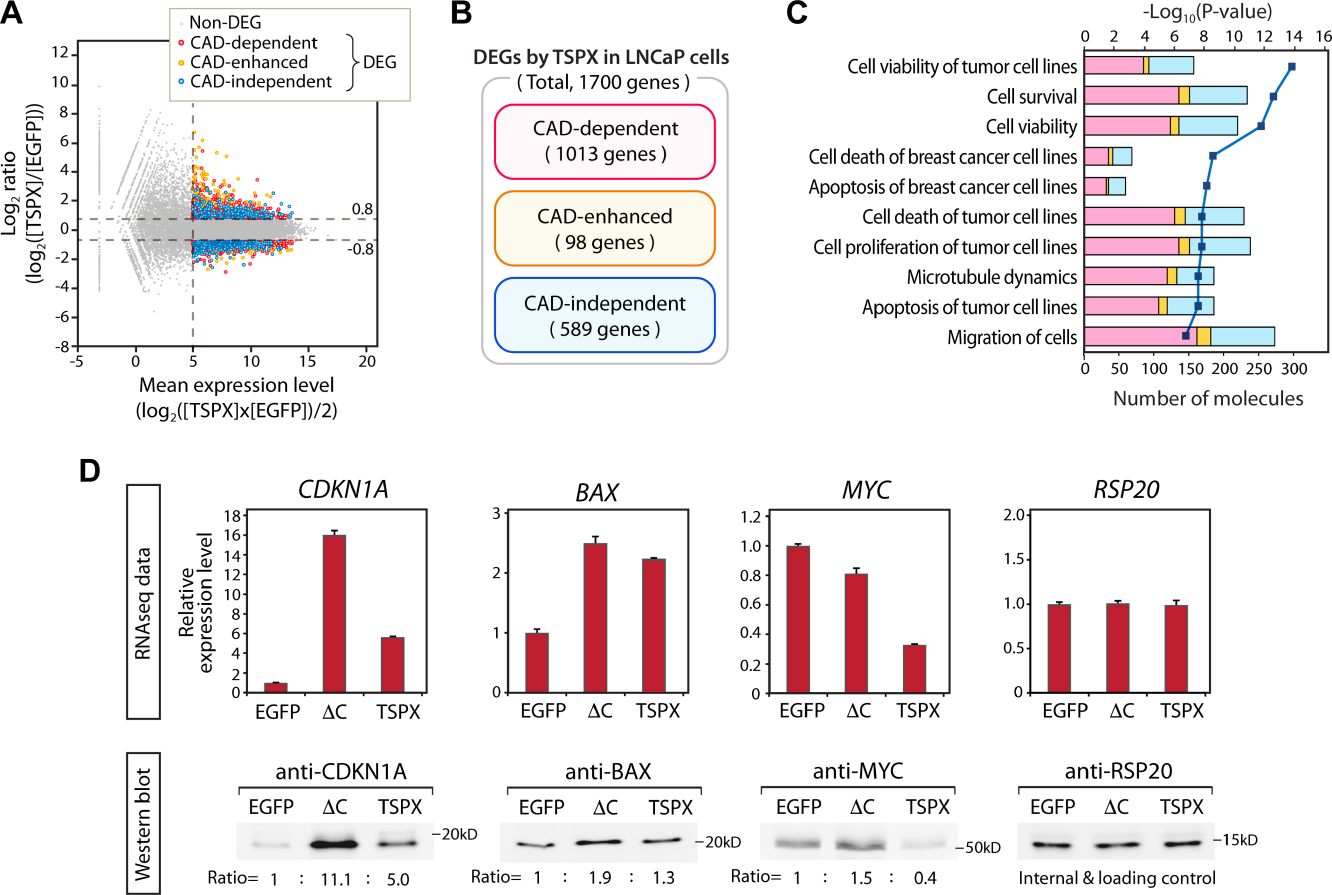
Comparative analysis of the transcriptomes of the LNCaP-tetON-TSPX and LNCaP-tetON-ΔC cells and identification of the CAD-dependent, CAD-enhanced, and CAD-independent differentially expressed genes (DEGs) (**A**) MA plots representing the gene expression changes mediated by TSPX-overexpression in LNCaP cells. Red plots indicate the CAD-dependent DEGs, orange plots indicate the DEGs whose expression changes were enhanced by CAD (CAD-enhanced), and blue plots indicate the CAD-independent DEGs. Gray plots indicate non-differentially expressed genes (non-DEG). (**B**) Venn diagram showing the number of genes classified into the CAD-dependent, CAD-enhanced, and CAD-independent DEGs, respectively. (**C**) Results of Ingenuity Pathway Analysis (IPA) for DEGs mediated by TSPX-overexpression in LNCaP cells. Top ten biological functions identified among the DEGs are presented. A dark blue line indicates -log_10_(*P*-value) and bars indicate the numbers of DEGs associated with respective pathways. Bar colors indicate CAD-dependent (red), CAD-enhanced (orange), and CAD-independent (blue) DEGs respectively. (**D**, top panel) The relative expression levels of CDKN1A, BAX, and MYC in the Dox-induced LNCaP-tetON-EGFP (EGFP), LNCaP-tetON-ΔC (ΔC), and LNCaP-tetON-TSPX (TSPX) cells. Expression values are presented based on the normalized RNA-Seq count data (*n* = 3) (mean ± SEM). RSP20 was shown as an internal control. (D, bottom panel) Western-blot confirmed the corresponding protein expression of CDKN1A, BAX, and MYC in the Dox-treated LNCaP-tetON-EGFP, LNCaP-tetON-ΔC, and LNCaP-tetON-TSPX cells. Ratio values indicate the relative band-intensity normalized to RSP20 respectively.

To explore the importance of CAD in TSPX functions in LNCaP cells, we performed RNA-Seq transcriptome analyses of both LNCaP-tetON-TSPX and LNCaP-tetON-ΔC cells with LNCaP-tetON-EGFP cells as controls using the Illumina RNA-Seq platform. The mRNAs were isolated from respective LNCaP cells in biological triplicates after Dox induction for 24 hours and subjected to RNA-Seq analysis. Genes representing changes with FDR< 0.001, Log_2_(gene expression level)>5, and |Log_2_(fold difference)|>0.8 were considered as differentially expressed genes (DEGs). The results showed that 1700 genes, including 818 upregulated genes and 882 downregulated genes, were differentially expressed by TSPX-overexpression (Figure 4A and Supplementary Table 1). There were 2514 DEGs induced by TSPX[ΔC] overexpression, consisting of 1037 upregulated and 1477 downregulated genes (Supplementary Table 1). To evaluate the effects of TSPX and the contribution of the CAD to its functions, the DEGs of the two groups were compared by plotting their differential expression levels (Supplementary Figure 1). There were 911 genes specifically affected by TSPX but not TSPX[ΔC], hereby designated as CAD-specific DEGs. There were 102 genes inversely affected by TSPX and TSPX[ΔC], i.e. the gene expression changes were inverted in TSPX and TSPX[ΔC]-expressing cells, hereby designated as CAD-inverted DEGs. In the present study, the CAD-specific DEGs and the CAD-inverted DEGs were combined and designated as the CAD-dependent DEGs for subsequent analyses, since CAD was critical for the gene expression changes of both groups. There were 98 genes affected by TSPX and to lesser extent by TSPX[ΔC], hereby designated as CAD-enhanced DEGs. There were 589 genes affected by TSPX[ΔC] and to lesser extent by TSPX, hereby designated as CAD-independent DEGs since the presence of the CAD in TSPX was not required for the changes (Figure 4A, 4B, and Supplementary Table 2). In addition, 832 genes were affected only by TSPX[ΔC] specifically but not TSPX, hereby designated as TSPX[ΔC]-specific DEGs.

To gain insights into the biological functions and pathways regulated by TSPX and the importance of the CAD in cellular properties associated with its overexpression in LNCaP cells, we had analyzed the CAD-dependent, CAD-enhanced and CAD-independent DEGs with the Ingenuity Pathways Analysis (IPA) [22, 35, 36]. The results showed that the biological functions involved in cell viability and cell death were prominently affected by TSPX-overexpression in LNCaP cells (Figure 4C and Supplementary Table 3). In addition, genes associated with microtubule dynamics and cell-migration were also affected (Figure 4C and Supplementary Table 3). Sixty to 70% genes of respective biological functions were affected by TSPX in a CAD-dependent manner (Figure 4C, red column) or a CAD-enhanced manner (Figure 4C, orange column), suggesting that CAD and possibly other domain(s) of TSPX might regulate respective genes involved in these functions and pathways. For example, the expression levels of tumor suppressor genes CDKN1A (also known as p21) and BAX were upregulated by TSPX[ΔC], indicating that these genes were upregulated in a CAD-independent manner (Figure 4D). In contrast, the oncogene MYC expression level was dramatically downregulated by TSPX (log_2_(fold change)= −1.609), while it was only slightly downregulated by TSPX[ΔC] (log_2_(fold change)= −0.370) (Figure 4D, top panels); indicating that the suppression of MYC expression was highly dependent on CAD. Western-blot analyses confirmed these results at the protein level, indicating that the CDKN1A and BAX proteins were increased by both TSPX[ΔC] and TSPX while the MYC protein was drastically decreased only by TSPX (Figure 4D, bottom panels). Although we cannot rule out any post-transcriptional modifications affected by TSPX or TSPX[ΔC], the correlation of changes between transcripts and proteins of most DEGs suggests that the TSPX regulatory functions are likely to be at the transcription levels.

### Correlation of the differentially expressed genes between the TSPX-overexpression in LNCaP cells and TSPX-high expression group of prostate cancer specimens

**Figure 5:**
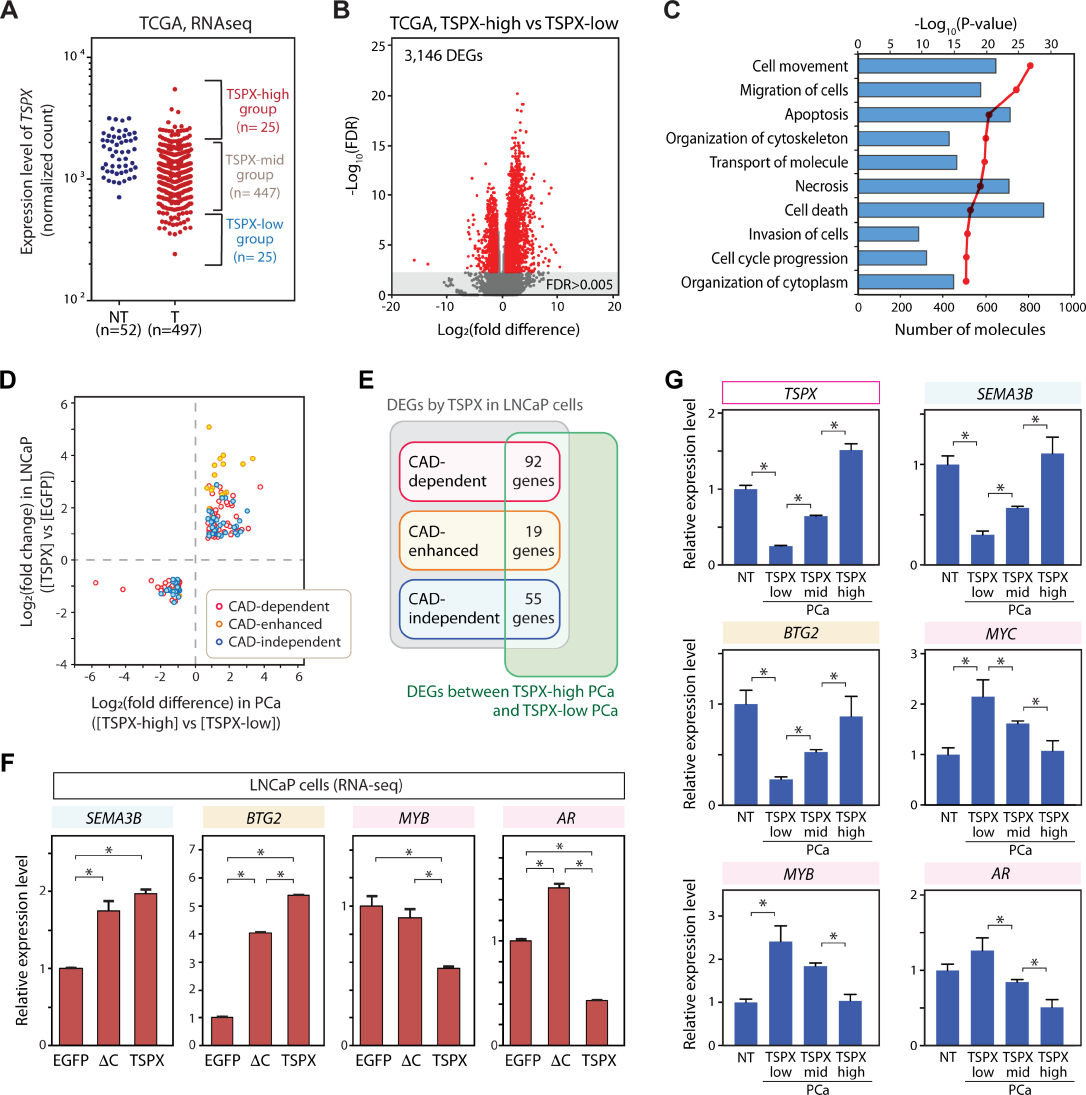
Identification of the consistent downstream genes of TSPX (TSPX-target genes) in both clinical prostate cancer samples and LNCaP cells (**A**) Based on the TSPX expression level, prostate cancer cases were classified into the TSPX-high group (highest 25 cases), the TSPX-low group (lowest 25 cases), and TSPX-mid group (the remainder of 447 cases) of 497 prostate cancer specimens. (**B**) Volcano plot representing the DEGs between TSPX-high and TSPX-low prostate cancer groups (red plots) with a FDR < 0.005. (**C**) Ingenuity Pathway Analysis (IPA) of DEGs between TSPX-high and TSPX-low expression level in prostate cancer. Top ten biological functions identified among the DEGs are presented. Red line indicates -log_10_(*P*-value) and bars indicate the numbers of DEGs associated with respective pathways. (**D**) A graph plotting the 166 TSPX-target genes whose expression levels (up or down) were consistently corresponded with those of TSPX in both clinical prostate cancer samples (X-axis) and LNCaP cells (Y-axis). Plot color indicates CAD-dependent (red), CAD-enhanced (orange), and CAD-independent (blue), respectively. (**E**) Venn diagram summarizing the number of CAD-dependent, CAD-enhanced, and CAD-independent TSPX-target genes. (**F**) The relative expression levels of SEMA3B, BTG2, MYB, and AR, in the Dox-induced LNCaP-tetON-EGFP, LNCaP-tetON-ΔC, and LNCaP-tetON-TSPX cells. Expression values are normalized RNA-Seq count data (*n* = 3) (mean ± SEM). Asterisks indicate the Student’s *t*-test *P*-value < 0.05. (**G**) The gene expression patterns of TSPX- SEMA3B, BTG2, MYB, and AR, in clinical samples. Relative expression levels in non-tumor prostate tissues (NT), TSPX-low prostate cancer (PCa) group, TSPX-mid PCa group, and TSPX-high PCa group. Asterisk indicates the Student’s *t*-test *P*-value < 0.05.

To correlate the effects of TSPX on cultured LNCaP cells to those of clinical prostate cancer, we had analyzed the transcriptomes of 497 clinical prostate cancer specimens from the TCGA database. The top 25 cases with the highest TSPX expression levels, hereby designated a TSPX-high group, were compared to those of 25 cases with the lowest TSPX expression levels, hereby designated as TSPX-low group (Figure 5A). The remainder of 447 cases were designated as TSPX-mid (middle expression level) group. The differentially expressed genes (DEGs) between TSPX-high and TSPX-low groups were selected with the criteria of false discovery rate FDR<0.005 and |Log_2_(fold difference)|>0.8 (Figure 5B and Supplementary Table 4). A total of 3146 DEGs between the two groups were identified and analyzed with IPA. Our results showed that the TSPX expression level might be associated with activities of various pathways of cell movement, cell death/apoptosis, and organization of cytoskeleton (Figure 5C and Supplementary Table 5). The association between the TSPX expression level and the pathways of cell death/apoptosis in clinical prostate cancer specimens was consistent with that in LNCaP cells (Figure 4C and 5C).

To identify the potential TSPX downstream gene in clinical prostate cancer, we had analyzed the differential gene expression patterns of LNCaP cells overexpressing TSPX versus EGFP (Figure 4A) and those of TSPX-high group versus TSPX-low group in clinical prostate cancer samples from TCGA datasets (Figure 5B). The DEGs with Student’s *t*-test *P*-value < 0.05 were used in the analysis to identify the downstream genes with stronger correlations. Our results showed that 166 DEGs were identified to share similar expression patterns associated with the TSPX expression level in both LNCaP cells and clinical prostate cancer specimens (Figure 5D and Supplementary Table 6). Out of these common 166 DEGs, 92 genes were CAD-dependent, 19 genes were CAD-enhanced, and 55 genes were CAD-independent DEGs (Figure 5E). Exploration by using CancerMine program [37] indicated many downregulated DEGs as caner-drivers/oncogenes, including MYC, MYB and AR (Supplementary Figure 2). Significantly, MYC, MYB and AR were downregulated by TSPX in a CAD-dependent manner in LNCaP cells (Figures 4D and 5F). In contrast, the upregulated DEGs included both caner-drivers/oncogenes, e.g. FGFR1 and MAP3K8, and tumor suppressors, e.g. SEMA3B and BTG2 (Supplementary Figure 2). SEMA3B and BTG2 were upregulated by TSPX in either CAD-independent or CAD-enhanced manner in LNCaP cells (Figure 5F). Interestingly, CDKN1A and BAX were upregulated by TSPX-overexpression in LNCaP cells (Figure 4D), but not in TSPX-high clinical prostate cancer samples (Supplementary Table 4). Since various factors are involved in regulations of CDKN1A and BAX genes respectively [38, 39], the effect(s) of TSPX on these genes could potentially be minimized by other factors and/or cancer heterogeneity.

To further explore the roles of TSPX in non-tumor prostate and the dosage dependent effects of TSPX in prostate cancer, the expression levels of tumor suppressors, SEMA3B and BTG2, and caner-drivers/oncogenes, MYC, MYB and AR, were compared among non-tumor prostate, TSPX-low prostate cancer, TSPX-mid prostate cancer, and TSPX-high prostate cancer samples. Our results showed that the expression levels of SEMA3B and BTG2 in the TSPX-high prostate cancer samples were similar with those in the non-tumor prostate samples (*P*-value > 0.05), while those in TSPX-low prostate cancer samples were significantly low (Figure 5G). On the other hand, the expression levels of MYC and MYB in TSPX-low prostate cancer samples were significantly higher than those in both non-tumor prostate samples and TSPX-high prostate cancer samples (Figure 5G). These results suggest that TSPX may play crucial roles to maintain the expressions of tumor suppressor genes, including SEMA3B and BTG2, and suppress oncogenes, such as MYC and MYB, in non-tumor prostate. Importantly, the expression levels of these genes were closely correlated with those of TSPX expression in the prostate cancer specimens, i.e. TSPX-low, TSPX-mid, and TSPX-high respectively (Figure 5G), suggesting that the regulatory effects of TSPX on its targets in prostate cancer could be dosage dependent. The expression level of AR was lower only in TSPX-high prostate cancer samples, and there was no significant difference between non-tumor prostate samples and TSPX-low prostate cancer samples (Figure 5G), suggesting that AR could serve an essential function(s) not affected by TSPX in normal prostatic tissues.

## DISCUSSION

TSPX has long been postulated as a tumor suppressor for lung cancer [17, 21], and numerous mutations have been demonstrated in various types of cancer [40, 41]. Although previous studies showed that it represses cell proliferation and negatively affects the cyclin B/CDK1 kinase activity and AR transactivation, its role(s) in prostate cancer has not been defined. The present study shows that the altered expression level of TSPX could be correlated with upregulation of various cancer-drivers/oncogenes and repressions of tumor suppressors in both LNCaP cell line and clinical prostate cancer samples in a dosage dependent manner (Figure 5 and Supplementary Table 6). Since overexpression of TSPX retards cell proliferation and induces cell death in LNCaP cells (Figure 2), elevated levels of TSPX expression could be important for its tumor suppressor function(s) in prostate cancer. For example, the expression levels of cancer-drivers/oncogenes MYC, MYB, and AR, whose roles in prostate cancer have been well established, were consistently lower in both TSPX-overexpressing LNCaP cells and TSPX-high clinical prostate cancer samples than the corresponding controls (Figure 5, and Supplementary Table 6). In particular, MYC is an oncogene involved in the initiation and progression of various types of cancer through its collaborative functions with other cancer-related genes [42, 43]. The high MYC expression level(s) is associated with poor clinical outcomes [44–46]. MYC is frequently upregulated in 70–90% of both prostatic intraepithelial neoplasia (PIN) and prostate cancer lesions, and the high-level MYC expression contributes to the initiation and progression of prostate cancer [47–50].

The prostate cancer susceptibility locus at Xp11.22 has been identified with the SNP rs5945572 by a genome-wide association study of 23,000 Icelanders and replicated with 15,500 European men [8] and American populations [51, 52]. At present, the exact nature of such susceptibility locus is still uncertain, although NUDT11, the gene most proximal to the rs5945572, has been implicated in such cancer predisposition [53]. As a tumor suppressor located at Xp11.22, TSPX could play a role in such cancer susceptibility. Similarly, another tumor suppressor gene, FOXP3, located at the Xp11.23 could also be involved. Knocking-down of FOXP3 results in an elevated MYC expression in human prostate epithelial cells (HPECs), and reactivation of FOXP3 expression downregulates MYC expression in prostate cancer cell lines [10]. Importantly, TSPX also downregulates MYC expression in both LNCaP cells (Figure 4D) and TSPX-high prostate cancer specimens (Figure 5G). Accordingly, the prostate cancer susceptibility locus at Xp11.2 could be related to the regulation of the MYC oncogene expression level. Inactivation and/or mutations of TSPX and/or FOXP3 tumor suppressors at the Xp11.2 locus might impair their regulatory functions on MYC expression, thereby exacerbating the susceptibility of prostate cancer initiation and progression.

In addition to MYC, TSPX also represses MYB, an oncogenic transcription activator involved in the development of various malignant tumors including leukemia, colon cancer, and breast cancer, as well as prostate cancer [54–57]. It is frequently upregulated in the metastatic prostate cancer and contributes to the castration resistance [55, 56]. Accordingly, TSPX-dependent suppression of MYB expression could constitute part of its tumor suppressor functions in castration resistant prostate cancer.

TSPX overexpression in LNCaP cells and TSPX-high clinical prostate specimens are associated with elevated expression levels of two tumor suppressors SEMA3B and BTG2 (Figure 5, and Supplementary Table 6). SEMA3B is a secreted member of the semaphorin family, originally identified as a molecule for axonal guidance. Recent studies have revealed that SEMA3B could be a tumor suppressor in various cancer types, including lung cancer, breast cancer, and esophageal squamous cell carcinoma [58, 59]. High levels of SEMA3B expression are associated with better survival of prostate cancer patients [60]. BTG2 (also known as PC3 or TIS21) is a member of TOB/BTG family participating in various anti-proliferative mechanisms [61–64]. BTG2 is frequently downregulated in prostate cancer, and its reduced expression is correlated with development of castration-resistant prostate cancer [65, 66], suggesting that BTG2 plays an important role in suppression of prostate cancer progression. Our findings, therefore, support the role of TSPX as a tumor suppressor for prostate cancer via its actions in repressing the expression of various cancer-drivers/oncogenes and stimulating those of tumor suppressors.

As discussed, TSPX and its Y chromosome homologue, TSPY, evolved from the same ancestral gene, but diverged in functions on the modern-day sex chromosomes [27, 30]. So far, we demonstrated that the C-terminal acidic domain (CAD) of TSPX plays important roles in its inhibitory actions on the cyclin B/CDK1 kinase activity and the transactivation function of AR [22, 28]. Using different variants of TSPX, we showed that the proline-rich domain plays minimal role(s) in regulation of cell proliferation. Overexpression of TSPX[ΔC], a CAD-truncated variant, was sufficient to arrest cell proliferation (Figure 3), while overexpression of TSPX could arrest cell proliferation and induce morphological/structural changes and eventually cell death (Figure 2). Comparative analyses of the transcriptomes of LNCaP-tetON-TSPX cells and LNCaP-tetON-ΔC cells revealed that a large number of the TSPX downstream genes were regulated in either CAD-dependent or CAD-enhanced manners, particularly pathway networks associated with cell viability and cell death in LNCaP cells (Figure 4). Importantly, the key cancer-drivers/oncogenes MYC, MYB and AR were suppressed by TSPX in a CAD-dependent manner (Figures 4 and 5). In addition, other cancer-drivers/oncogenes, e.g. IGF1, CDK6 and PDK1 that are widely involved in oncogenesis [67–69], were also suppressed by TSPX in a CAD-dependent manner (Supplementary Figure 2 and Supplementary Table 2). These results strongly suggest that CAD is important for the tumor suppressor functions of TSPX at transcriptional regulation, i.e. downregulating various downstream cancer-drivers/oncogenes and stimulating other tumor suppressors.

**Figure 6:**
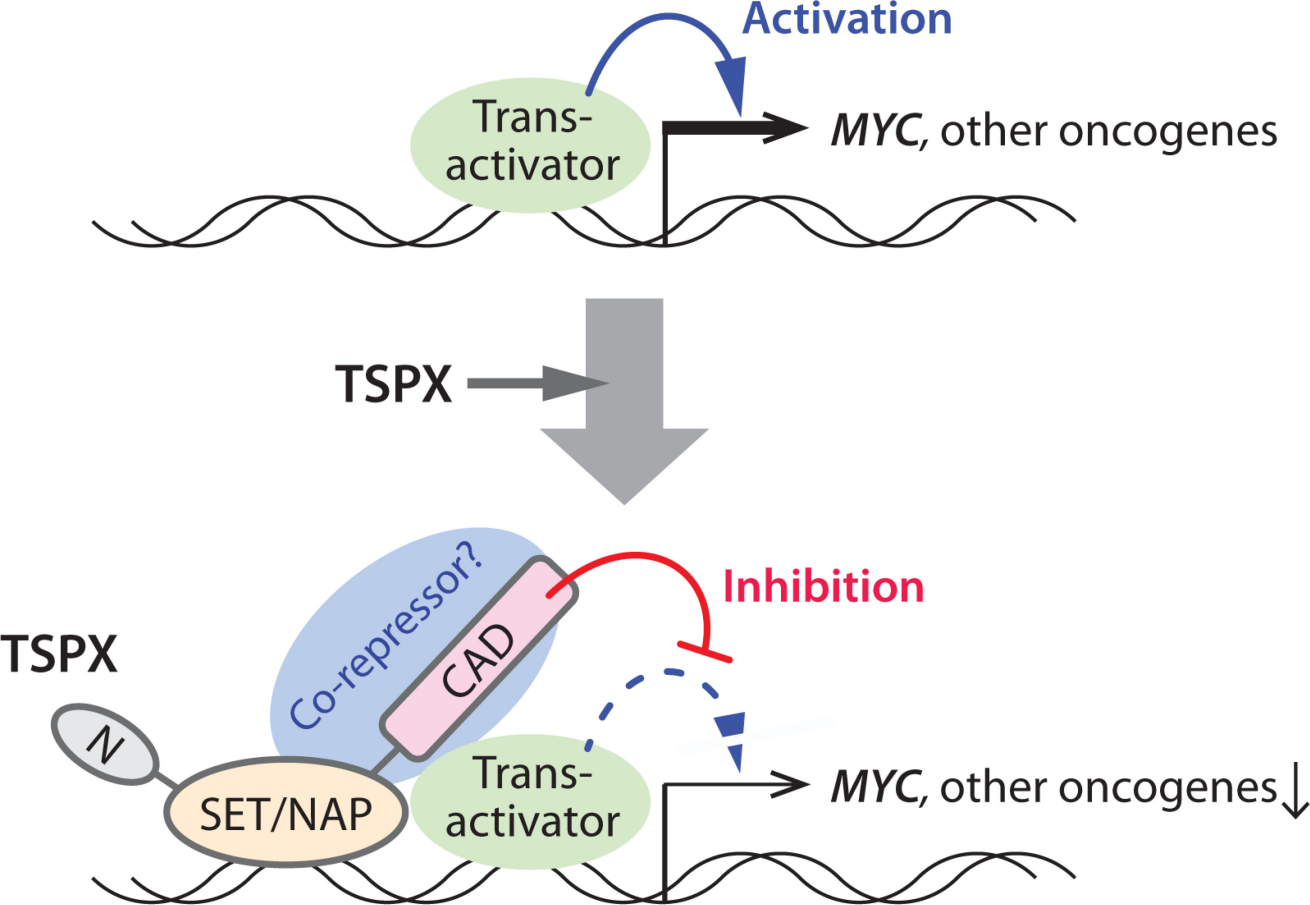
A schematic diagram illustrating the likely mechanism(s) of the TSPX-mediated suppression of the MYC gene TSPX interacts with the MYC promoter region via its SET/NAP domain, and the C-terminal acidic domain (CAD) plays an important role to suppress the MYC gene, likely by recruiting co-repressors to the promoter. Other cancer-drivers/oncogenes that were downregulated by TSPX may be also regulated in the similar manner(s).

At present, the exact mechanism(s) of TSPX transcriptional regulation is still uncertain. Previously, we showed that TSPX interacts with AR and represses the AR transactivation of target genes [22]. Additional studies demonstrated that TSPX is an essential component of the REST/NRSF repressor complex, involved in transcriptional regulation of genes [21]. Further, it binds to the promoters of neuronal genes and regulates their expressions via histone modification in primary mouse neurons [70]. Although there would likely be different target genes between neurons and cancer cells, TSPX may be able to exert its transcriptional regulatory functions via directly interaction and modulation on the promoter areas of its target genes (Figure 6). Indeed, a preliminary chromatin-immunoprecipitation (ChIP)-PCR assay of the Dox-induced LNCaP-tetON-TSPX cells and LNCaP-tetON-ΔC cells showed that both TSPX and TSPX[ΔC] could bind to the MYC gene promoter, thereby confirming its direct bindings to the genes being regulated by TSPX (Supplementary Figure 3).

Prostate cancer is highly heterogeneous cancer with significant variation in disease progression and mortality rate among the patients [71–73]. Hence, the clinical management of prostate cancer is still challenging in medicine [71, 72]. The present study demonstrates the importance of the CAD of TSPX in its tumor suppressor functions in prostate cancer and highlights the needs for further studies on TSPX-mediated gene regulation, thereby providing novel insights in diagnosis and prognosis for clinical treatment and management of this common cancer in men.

## MATERIALS AND METHODS

### Quantitative reverse-transcription polymerase chain reaction analysis (qRT-PCR) of human prostate tissue samples

Snap frozen tissue samples of human prostate cancer (prostate cancer) and adjacent non-tumorous prostate were obtained from the Cooperative Human Tissue Network (CHTN) (http://www.chtn.nci.nih.gov/). Total RNA was extracted from 15 pairs of prostate cancer and corresponding adjacent non-tumorous prostate using Trizol reagent (Invitrogen, Carlsbad, CA), according to the manufacturer’s instructions. After RQ1-DNase treatment (Promega, Madison, WI) to remove any contaminant DNA, cDNA was synthesized from 1 μg total RNA using the Transcriptor Reverse Transcriptase kit with oligo(dT)_15_ primer (Roche Applied Science, Indianapolis, IN). The synthesized cDNA samples were individually analyzed in triplicates by quantitative real-time PCR (qPCR) using the GoTaq qPCR Master Mix (Promega) and MyiQ real-time PCR detection system (Bio-Rad, Hercules, CA) as described previously [19]. The primer sequences were; glyceraldehyde 3–phosphate dehydrogenase (GAPDH) primers, 5′ -CCACCCATGGCAAATTCCATGGCA-3′ and 5′ -TCTAGACGGCAGGTCAGGTCCACC-3′; TSPX primers, 5′-ACTTACGGCAACAACTTCTTCAAA-3′ and 5′- AGGGCAAATGGGATAGTGGGTG-3′. The TSPX gene expression levels were normalized based on the corresponding levels of GAPDH in the same samples, and differential expression levels between tumor and non-tumorous sample pairs was then calculated and further analyzed with the Student’s *t*-test. Any differential expression level with *P*-value < 0.05 was considered to be statistically significant. The expression level in cultured cell lines, LNCaP, DU145, and PC3, were measured similarly.

The human study was performed under an exempt protocol approved by the Institutional Committee on Human Research, VA Medical Center, San Francisco.

### Plasmids and lentiviruses

Several TSPX variants were used in the present study: an isoform harboring the longest N-terminal region, designated as TSPX[FL] (also called CDA1); a variant harboring shorter N-terminal segment, designated as TSPX, and a variant with a deleted CAD (397-693aa), designated as TSPX[ΔC] were used (Figure 2C) [34, 74, 75]. The DNA fragments coding TSPX[FL] and FLAG epitope-tagged TSPX variants were extracted from the previously described plasmids, e.g. pcDNA3.1-TSPX[full], p3xFLAG-TSPX[ΔPro] and p3xFLAG-TSPX[ΔProΔC] [34], and inserted into pIRES2-EGFP vector (Clontech/Takara bio, Mountain View, CA), thereby adding a co-expressed EGFP at the 3’-end of the respective TSPX coding sequence. The bicistronic cassettes of TSPX-ires-EGFP were inserted into the FUW-tetO vector, respectively [76] (Addgene, Cambridge, MA), resulting in various FUW-tetO-TSPX expression vectors capable of expressing the respective TSPX variants and EGFP under the control of doxycycline (Dox) (Figure 2B). FUW-tetO-EGFP was used as a negative control. The generation of replication-incompetent lentiviruses followed the methods previously reported [22, 76].

### Cell culture

The human prostate adenocarcinoma cell lines, LNCaP, PC3 and DU145 were purchased from the American Type Culture Collections (ATCC, Manassas, VA) and cultured in RPMI 1640 medium containing 10% fetal bovine serum (FBS, Clontech) and antibiotics cocktail (100 U/mL penicillin and 100 μg/mL Streptomycin; HyClone/GE Healthcare Life Science). For doxycycline induction, LNCaP cells were cultured in the medium supplemented with tet system-approved fetal bovine serum (tetracycline-free FBS, Clontech) and simultaneously transduced with lentiviral vectors, FUW-tetO-EGFP or FUW-tetO-TSPX variants and the transactivator, FUW-M2rtTA. The transduced cells were cultured under non-induced conditions, in the absence of doxycycline (Dox). To induce the expression of TSPX and/or EGFP in the transduced cells, cells were cultured in the presence of 1 μg/mL Dox (Sigma-Aldrich). For cell proliferation analyses, cells were seeded at 2×10^3^ cells/well in 96 well plates and cultured in the presence or absence of 1 μg/mL Dox. The cell viability was monitored at the indicated time points by using the CellTiter 96 Aqueous One Cell Proliferation Assay kit (Promega), a 3-(4,5-dimethylthiazol-2-yl)-5-(3-carboxymethoxyphenyl)-2-(4-sul-fophenyl)-2H-tetrazolium (MTS) based assay system, according to the manufacturer’s instructions.

### Western-blot

Western-blot was performed as described previously [34], using anti-FLAG mouse monoclonal IgG (clone M2, Sigma-Aldrich), anti-MYC rabbit monoclonal IgG (clone Y69, Abcam), anti-CDKN1A rabbit monoclonal IgG (clone EPR362, Abcam), anti-BAX rabbit monoclonal IgG (clone E63, Abcam), anti-RPS20 rabbit monoclonal IgG (clone EPR8716, Abcam), anti-βactin mouse monoclonal IgG (clone AC-15, Sigma-Aldrich), and anti-TSPX rabbit IgG (against C-terminal region; Proteintech, Rosemont, IL). Immunoreactive signals were visualized by IRDye680RD conjugated anti-mouse IgG antibody or IRDye800CW conjugated anti-rabbit IgG antibody, and scanned by Odyssey system (LI-COR, Lincoln, NE). The band intensity was analyzed by using ImageJ software [77] (https://imagej.nih.gov/ij/).

### Immunofluorescence

Immunofluorescence was performed as described previously [78]. Briefly, cells were fixed by 4% paraformaldehyde-PBS solution for 5 min, and permeabilized by methanol treatment. After blocking by 3% bovine serum albumin (Sigma)-PBS solution for 1 hr, slides were incubated with primary antibodies at 4° C overnight. The primary antibodies used for immunofluorescence were anti-GFP goat IgG (Abcam) and anti-FLAG tag mouse monoclonal IgG. The immunoreactive signals were visualized by Alexa Fluor 594 conjugated anti-mouse IgG antibody and Alexa Fluor 488 conjugated anti-goat IgG antibody (Invitorgen). Nuclear DNA was visualized by staining with 4’,6-diamidino-2-phenylindole (DAPI) (Roche Applied Science). Fluorescent images of the living cells and immunofluorescence were recorded with a Nikon Eclipse Ti digital camera and image acquisition workstation (Nikon instrument Inc., Melville, NY).

### BrdU incorporation assay

Cells were seeded at 1.6 × 10^4^ cells/well on 4 well chamber slides (Nunc Lab-Tek II CC2, Fisher Scientific) and cultured in the absence of Dox for 24 hours. Doxycycline at 1 μg/mL was then added in the medium to induce transgene expression. At 24 hours post-induction, cells were cultured with 10 μM 5-Bromo-2’-deoxyuridine (BrdU) (Sigma-Aldrich) for 2 hours, and fixed by 4% paraformaldehyde for 5 min. The cells labeled by BrdU were detected by immunofluorescence using anti-BrdU mouse monoclonal IgG (clone BU-1; GE Healthcare Life Sciences) as described above.

### Annexin-V binding assay

Apoptotic and dead cells in culture were detected by Annexin-V binding assay using Alexa Fluor 594 conjugated Annexin-V (Invitrogen) and Annexin-V binding buffer (Invitrogen) according to manufacturer’s instructions. Fluorescent images were recorded as describe above.

### Scratch test

The migratory properties and morphological changes were measured by a scratch wound assay [79]. Briefly, cells were plated at 1×10^6^ cells/10 cm dish and cultured for 48 hours in the absence of Dox. Before treatment with Dox, dishes were scratched with a 1000 μl pipette tip. Cells were cultured with fresh medium with or without 1 μg/mL Dox. Images of the scratched areas were recorded at 0, 24, 48, and 72 hours after scratching.

### RNA preparation and RNA-Seq transcriptome analysis for LNCaP cells

Total RNA was isolated from the LNCaP cells cultured in 6-well plates (9.6 cm^2^ surface area) at 24 hours after Dox-induction using the TRIZOL-Plus RNA purification kit (Amnion/Thermo Fisher Scientific, NY). Each group was analyzed with biological triplicates with 1 μg total RNA each. Combined mRNA purification and cDNA library preparations were performed with the KAPA Stranded mRNA-Seq kit (Kapa Biosystems, MA). Libraries were indexed with the NEBNext multiplex oligos for Illumina (New England Biolabs, CA). The libraries were subjected to 75-single end read cycles of sequencing on the NextSeq 500 (Illumina, CA). All procedures were performed according to the manufacturer’s instructions.

After quality assessment by FastQC program [80], the sequence reads were mapped onto the human reference genome GRCh37/hg19 by using STAR [81]. The mapped reads were summarized and calculated to the count reads that could be associated with the expression levels using the featureCounts program [82]. Normalization of data and differential gene expression analysis were performed using TCC/edgeR software package [83].

### Data source and data mining procedures

Gene expression data (level 3 RNA-Seq version 2) of prostate adenocarcinoma were downloaded from the Cancer Genome Atlas (TCGA) portal (http://cancergenome.nih.gov), which included data of 52 tumor and non-tumor paired samples and 445 unpaired tumor samples, 497 prostate cancer samples in total. Statistical analyses were performed using Prism6 program (GraphPad Software, Inc., La Jolla, CA) and Excel (Microsoft, Redmond, WA). Differentially expressed genes (DEGs) between two classified groups were identified using TCC/edgeR software package [83].

Gene expression enrichment analysis, functional network analyses and pathway analyses were performed by using Ingenuity Pathways Analysis (IPA) (build version 463341M, QIAGEN) [22, 35, 36].

## SUPPLEMENTARY MATERIALS FIGURES AND TABLES














