# Supplementary material for: The X-linked tumor suppressor TSPX downregulates cancer-drivers/oncogenes in prostate cancer in a C-terminal acidic domain dependent manner
Source: Oncotarget. 2019 Feb 19;10(15):1491–506. doi: 10.18632/oncotarget.26673 (PMC6407674; doi:10.18632/oncotarget.26673)
Supplement: Supplementary file 1 [file oncotarget-10-1491-s001.pdf]

The X-linked tumor suppressor TSPX downregulates cancer-drivers/oncogenes in prostate cancer in a C-terminal acidic domain dependent manner

SUPPLEMENTARY MATERIALS

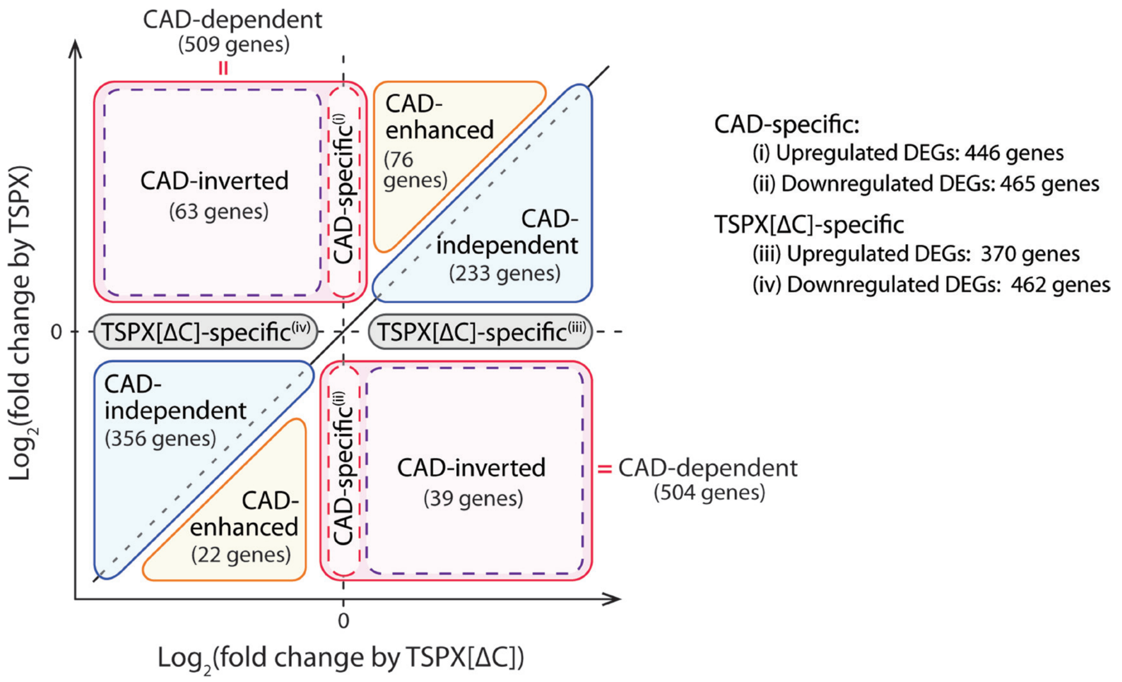

Supplementary Figure 1: Comparative analysis of the differentially expressed genes (DEGs) induced by TSPX (Y-axis) and TSPX[ΔC] (X-axis) overexpression in LNCaP cells. DEGs were classified into CAD-specific (dotted red area, specifically affected by TSPX), CAD-inverted (dotted purple area, differentially affected in the opposite directions by TSPX and TSPX[ΔC]), CAD-enhanced (solid orange area, affected by both TSPX and TSPX[ΔC] in the same direction), CAD-independent (solid blue area, not affected by TSPX), and TSPX[ΔC]-specific (solid gray area, specifically affected by TSPX[ΔC]). The CAD-specific DEGs and the CAD-inverted DEGSs were combined into a group, hereby designated as the CAD-dependent DEGs (solid red area) in the present study. Numbers of DEGs of respective classes are indicated.

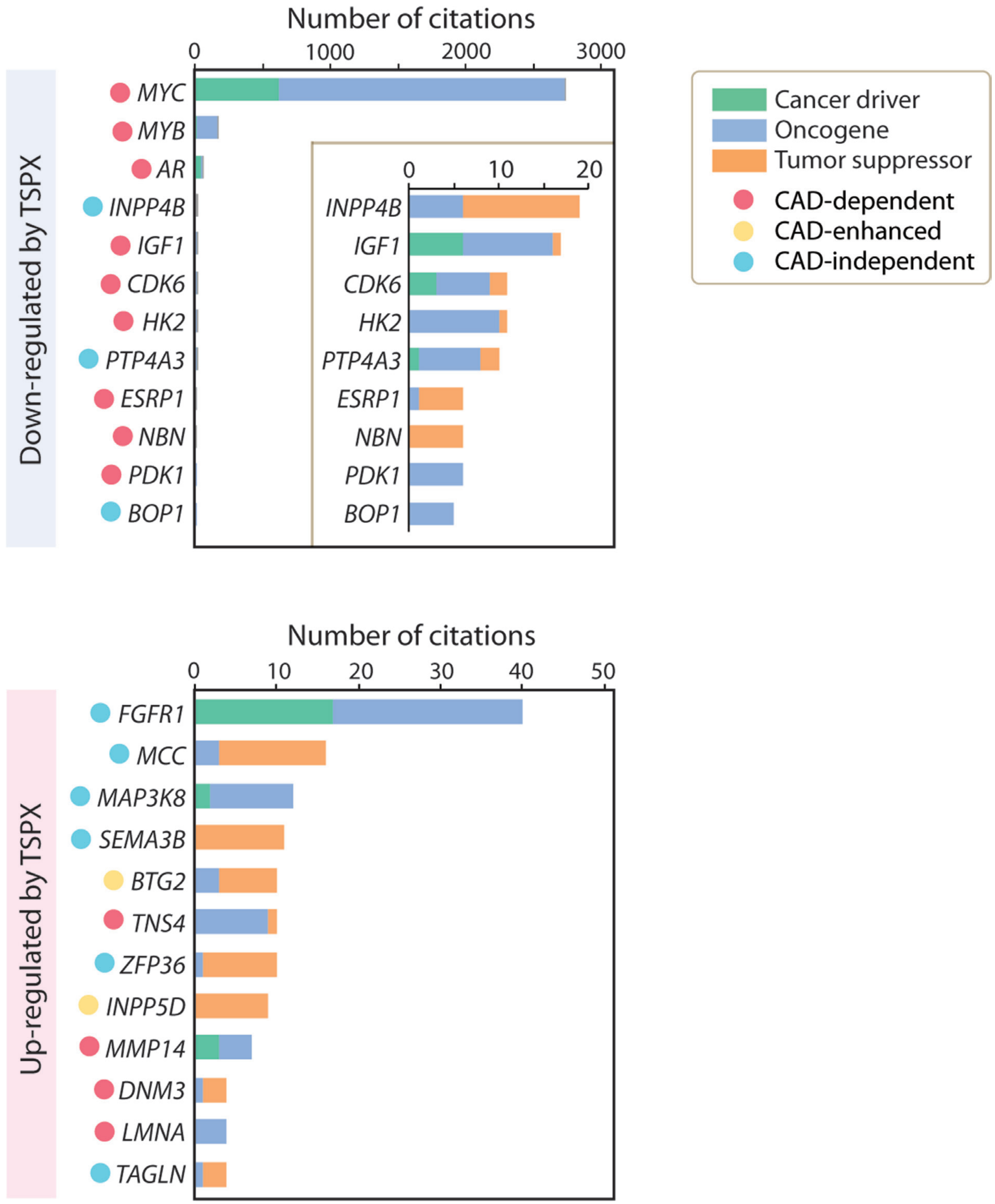

Supplementary Figure 2: TSPX affects the expressions of oncogenes/cancer-drivers and tumor suppressors. To explore the possible roles of DEGs commonly correlated with the TSPX-expression level in both LNCaP cells and clinical prostate cancer samples (Figure 5A and Supplementary Table 6), DEGs were analyzed by using the CancerMine program that analyzes the number of citations reporting the respective cancer-related functions, e.g. function of cancer-driver, oncogene, or tumor suppressor [1]. The number of citations reflects the importance of each molecule in cancer, and the DEGs with more than 4 citations were considered as the noticeable genes in the present study.

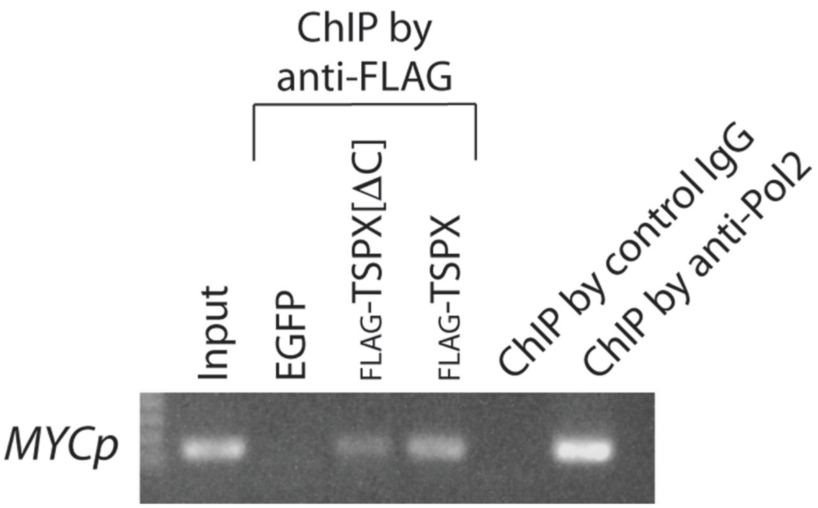

Supplementary Figure 3: TSPX binds to the MYC gene promoter. Chromatin-immunoprecipitation (ChIP)-PCR assay was used to demonstrate the bindings of TSPX and TSPX[ΔC] on the MYC gene promoter region in respectively overexpressed LNCaP cells, as described previously [2]. In brief, after 24 hr of Dox-induction, the respectively transduced LNCaP cells were processed for ChIP assay using EZ magna ChIP kit (MilliporeSigma, Burlington, MA) with anti-FLAG mouse IgG (clone M2, Sigma-Aldrich), according to the manufacturer's instructions. ChIPed DNA samples were analyzed by PCR (ChIP-PCR) using a primer set to detect the MYC gene promoter region; MYCp-1F, 5′-AGGGTTTGAGAGGGAGCAAA-3′, and MYCp-1R, 5′-GCATTATAAAGGGCCGGTGG-3′ (-20bp upstream from the transcription start site). The results showed that both TSPX[ΔC] and TSPX interacted with the *MYC* promoter region, suggesting that TSPX likely interacts with the *MYC* promoter via its SET/NAP domain present in both proteins independent of its CAD. Similar ChIP-PCR analyses with anti-RNA polymerase II mouse IgG (Millipore) (positive control) or negative control mouse IgG (1 µg/reaction) showed positive and negative signal respectively.

REFFERENCES

1. Lever J, Zhao EY, Grewal J, Jones MR, Jones SJM. CancerMine: A literature-mined resource for drivers, oncogenes and tumor suppressors in cancer. bioRxiv. 2018 Jul 09. https://doi.org/10.1101/364406 [Epub ahead of print]
2. Kido T, Lau YF. The Y-located gonadoblastoma gene TSPY amplifies its own expression through a positive feedback loop in prostate cancer cells. Biochem Biophys Res Commun. 2014; 446:206–11.

Supplementary Table 1: Differentially expressed genes (DEGs) between LNCaP-tstON-TSPX and LNCaP-tetON-EGFP, LNCaP-tetON-ΔC and LNCaP-tetON-EGFP, or LNCaP-tstON-TSPX and LNCaP-tetON-ΔC. See Supplementary_Table_1

Supplementary Table 2: Differentially expressed genes (DEGs) between LNCaP-tetON-TSPX and LNCaP-tetON-EGFP, or LNCaP-tetON-ΔC and LNCaP-tetON-EGFP, and the classification of DEGs based on the CAD dependency. See Supplementary_Table_2

Supplementary Table 3: Results of IPA biological function analysis for the differentially expressed genes (DEGs) between LNCaP-tetON-TSPX cells and LNCaP-tetON-EGFP cells at 24 hours after Dox-induction. See Supplementary_Table_3

Supplementary Table 4: Differentially expressed genes (DEGs) between TSPX-high PCa and TSPX-low PCa in TCGA datasets. See Supplementary_Table_4

Supplementary Table 5: Results of IPA biological function analysis for the differentially expressed genes between TSPX-high group and TSPX-low group prostate cancers in TCGA datasets. See Supplementary_Table_5

Supplementary Table 6: Consistent DEGs associating with the TSPX expression level in both clinical PCa specimens and LNCaP cells. See Supplementary_Table_6
